# Characterization of the complete chloroplast genome of *Salvia tiliifolia* Vahl (Lamiaceae)

**DOI:** 10.1080/23802359.2020.1768943

**Published:** 2020-05-27

**Authors:** Jing Wang, Danping Feng, Jun Qian, Baozhong Duan, Min Fan

**Affiliations:** a College of Pharmaceutical Science, Dali University, Dali, China; b Key Laboratory of Yunnan Provincial Higher Education Institute for Development of Yunnan Daodi Medicinal Materials Resources, Dali, China

**Keywords:** *Salvia tiliifolia*, complete chloroplast genome, phylogenetic analysis

## Abstract

*Salvia tiliifolia* Vahl is native to Central America and considered as an invasive plant in Mexico, the United States, Ethiopia, South Africa, and Australia. The complete chloroplast (cp) genome of *S. tiliifolia* was 150,836 bp in length, which contained a large single-copy (LSC) region of 82,129 bp, a small single-copy (SSC) region of 17,533 bp, and a pair of inverted repeats (IRs) region of 25,587 bp each. The genome harbored 130 genes, including 85 protein coding genes, 37 tRNA genes, and 8 ribosomal RNA genes. The overall GC content was 37.99%. Phylogenetic analysis indicated that *S. tiliifolia* is closely related to the species of *Salvia chanryoenica*.

*Salvia tiliifolia* Vahl (Lamiaceae) is an annual herb native to Central America and considered as an invasive plant in Mexico, the United States, Ethiopia, South Africa, and Australia (Hu et al. 2013). This species had been incorrectly identified as *Salvia dugesii* (a synonym for *Salvia melissodora*) in China (Xu et al. 2004). It is mainly distributed in west of Guizhou, southeast of Tibet, west of Sichuan, and all municipalities/autonomous prefectures of Yunnan Province (Hu et al. 2013). The aerial parts of *S. tiliifolia* were used in folk medicine for the treatment of diarrhea and neurodegenerative diseases (Tene et al. 2007; Adewusi et al. 2011). Until now, most of the studies on *S. tiliifolia* were focused on its chemical compositions and biological characteristics (Fan et al. 2017; González-Chávez et al. 2018). However, the complete chloroplast (cp) genome sequence of *S. tiliifolia* has not been reported so far. In this study, its cp genome was successfully assembled and annotated, and its relationship with closely related species was investigated.

Molecular materials of *S. tiliifolia* were collected from Dali county, Yunnan, China (25°71′99′′N, 100°26′02′′E), and the voucher specimen was deposited in the Herbarium of Dali University (LJ2020010511). Total DNA was isolated using the DNeasy plant mini kit (QIAGEN). Next-generation sequencing was carried out by an Illumina NovaSeq system (San Diego, CA). About 5.6 Gb of raw data (40,013,252 reads) was assembled by NOVOPlasty (Park et al. 2019; Liu et al. 2020), and the assembled cp genome was annotated by GeSeq with default sets (Tillich et al. 2017; Yang et al. 2020). The annotated cp genome was submitted to the GenBank with the accession number of MT381946.

The cp genome sequence of *S. tiliifolia* was 150,836 bp in length. with a large single-copy region (LSC) of 82,129 bp, a small single-copy region (SSC) of 17,533 bp, and a pair of inverted repeats (IR) regions of 25,587 bp each. A total of 130 genes were annotated in *S. tiliifolia* cp genome, including 85 protein-coding genes, 37 tRNA genes, and 8 ribosomal RNA genes. The overall GC content was 37.99%. In order to clarify the position of *S. tiliifolia* in the phylogenetic tree, a total of 16 cp genome sequences from related species in Lamiaceae were downloaded from the NCBI database and were aligned using MAFFT v7.307 (Katoh and Standley 2013; Liang et al. 2020). A neighbor-joining (NJ) tree with 1000 bootstraps was inferred using MEGA version 6.0 (Tamura et al. 2013), with *Dracocephalum palmatum* (NC 031874) as outgroup. The phylogenetic analysis showed that *S. tiliifolia* is closely related to the species of *Salvia chanryoenica* (Figure 1). The present study afforded scientific evidence for resource development of *S. tiliifolia* and would be beneficial to taxonomy and phylogeny of Lamiaceae.

**Figure 1.**
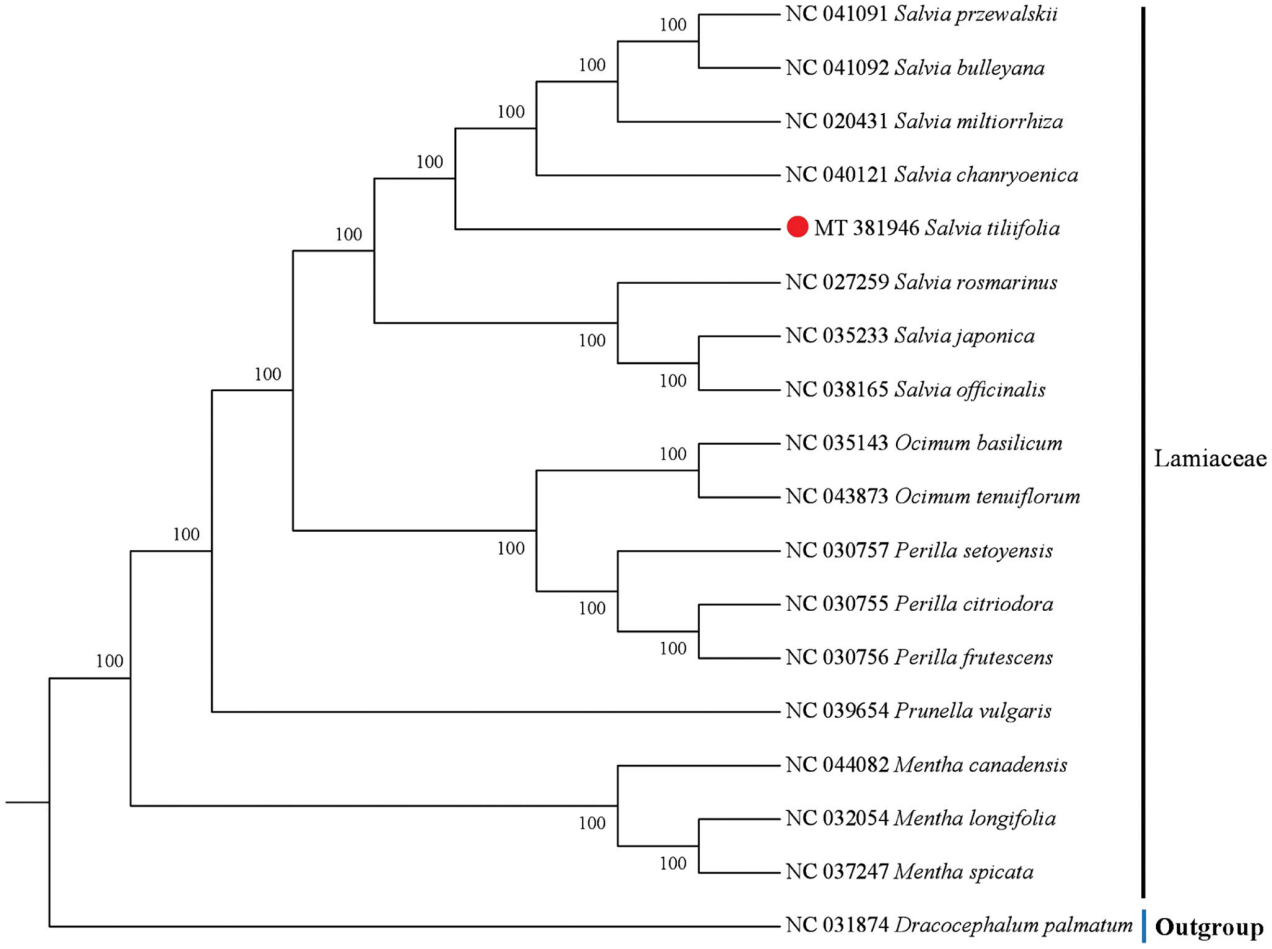
Neighbor-joining (NJ) tree based on the cp genome of 16 species of Lamiaceae with *Dracocephalum palmatum* as outgroup.

## Data Availability

The data that support the findings of this study are openly available in NCBI GenBank database at (https://www.ncbi.nlm.nih.gov) with the accession number is MT381946, which permits unrestricted use, distribution, and reproduction in any medium, provided the original work is properly cited.
